# Advancing athletic assessment by integrating conventional methods with cutting-edge biomedical technologies for comprehensive performance, wellness, and longevity insights

**DOI:** 10.3389/fspor.2023.1327792

**Published:** 2024-01-08

**Authors:** Marios Spanakis, Persefoni Fragkiadaki, Elisavet Renieri, Elena Vakonaki, Irene Fragkiadoulaki, Athanasios Alegakis, Mixalis Kiriakakis, Nikolaos Panagiotou, Eleni Ntoumou, Ioannis Gratsias, Evangelos Zoubaneas, Galina Dmitrievna Morozova, Marina Alekseevna Ovchinnikova, Christina Tsitsimpikou, Konstantinos Tsarouhas, Nikolaos Drakoulis, Anatoly Viktorovich Skalny, Aristides Tsatsakis

**Affiliations:** ^1^ Department of Forensic Sciences and Toxicology, School of Medicine, University of Crete, Heraklion, Greece; ^2^ Computational Bio-Medicine Laboratory, Institute of Computer Science, Foundation for Research and Technology – Hellas, Heraklion, Greece; ^3^ LifePlus Diagnostic & Consulting Health Services, Science Technology Park of Crete, Heraklion, Greece; ^4^ iDNA Laboratories, Athens, Greece; ^5^ Check Up Medicus Biopathology & Ultrasound Diagnostic Center – Polyclinic, Athens, Greece; ^6^ Diatrofi Center for Eating Disorders and Consultation, Athens, Greece; ^7^ Bioelementology and Human Ecology Center, I.M. Sechenov First Moscow State Medical University (Sechenov University), Moscow, Russia; ^8^ Department of Sport Medicine and Medical Rehabilitation, I.M. Sechenov First Moscow State Medical University (Sechenov Univercity), Moscow, Russia; ^9^ General Chemical State Laboratory of Greece, Athens, Greece; ^10^ Department of Cardiology, University Hospital of Larissa, Larissa, Greece; ^11^ Research Group of Clinical Pharmacology and Pharmacogenomics, Faculty of Pharmacy, School of Health Sciences, National and Kapodistrian University of Athens, Athens, Greece; ^12^ Medical Elementology Department, Peoples Friendship University of Russia, Moscow, Russia

**Keywords:** sports medicine, telomeres, genotype, metabolomics, nutrition, essential elements, echocardiography, athletic performance

## Abstract

In modern athlete assessment, the integration of conventional biochemical and ergophysiologic monitoring with innovative methods like telomere analysis, genotyping/phenotypic profiling, and metabolomics has the potential to offer a comprehensive understanding of athletes' performance and potential longevity. Telomeres provide insights into cellular functioning, aging, and adaptation and elucidate the effects of training on cellular health. Genotype/phenotype analysis explores genetic variations associated with athletic performance, injury predisposition, and recovery needs, enabling personalization of training plans and interventions. Metabolomics especially focusing on low-molecular weight metabolites, reveal metabolic pathways and responses to exercise. Biochemical tests assess key biomarkers related to energy metabolism, inflammation, and recovery. Essential elements depict the micronutrient status of the individual, which is critical for optimal performance. Echocardiography provides detailed monitoring of cardiac structure and function, while burnout testing evaluates psychological stress, fatigue, and readiness for optimal performance. By integrating this scientific testing battery, a multidimensional understanding of athlete health status can be achieved, leading to personalized interventions in training, nutrition, supplementation, injury prevention, and mental wellness support. This scientifically rigorous approach hereby presented holds significant potential for improving athletic performance and longevity through evidence-based, individualized interventions, contributing to advances in the field of sports performance optimization.

## 1 Introduction

Athletes and their coaches constantly aim to push the limits of their abilities, strive for ideal performance, surpass previous achievements, and sustain longevity (1). Comprehending an athlete's biochemistry, nutrition, and physiological processes is key to unlocking full athletic potential (2). Especially nowadays, with sufficient evidence of how environment and lifestyle affect overall health, biomedical advancements open a new frontier: integrating knowledge for improved wellness and longevity (3). For instance, genetic variations, epigenetic traits, and phenotypic expression have been related to pathophysiological characteristics and longevity (4). Extending it to metabolomics it offers a deeper understanding of a person's metabolic health (5). This knowledge can guide nutritional strategies considering energy needs and micronutrients (6). Non-invasive medical imaging techniques offer a detailed assessment of organs' structure and function that are crucial for medical monitoring (7). The thorough understanding gained from these techniques can facilitate evidence-based predictions along with preventive, participatory and personalized interventions for a person (8).

**Figure 1:**
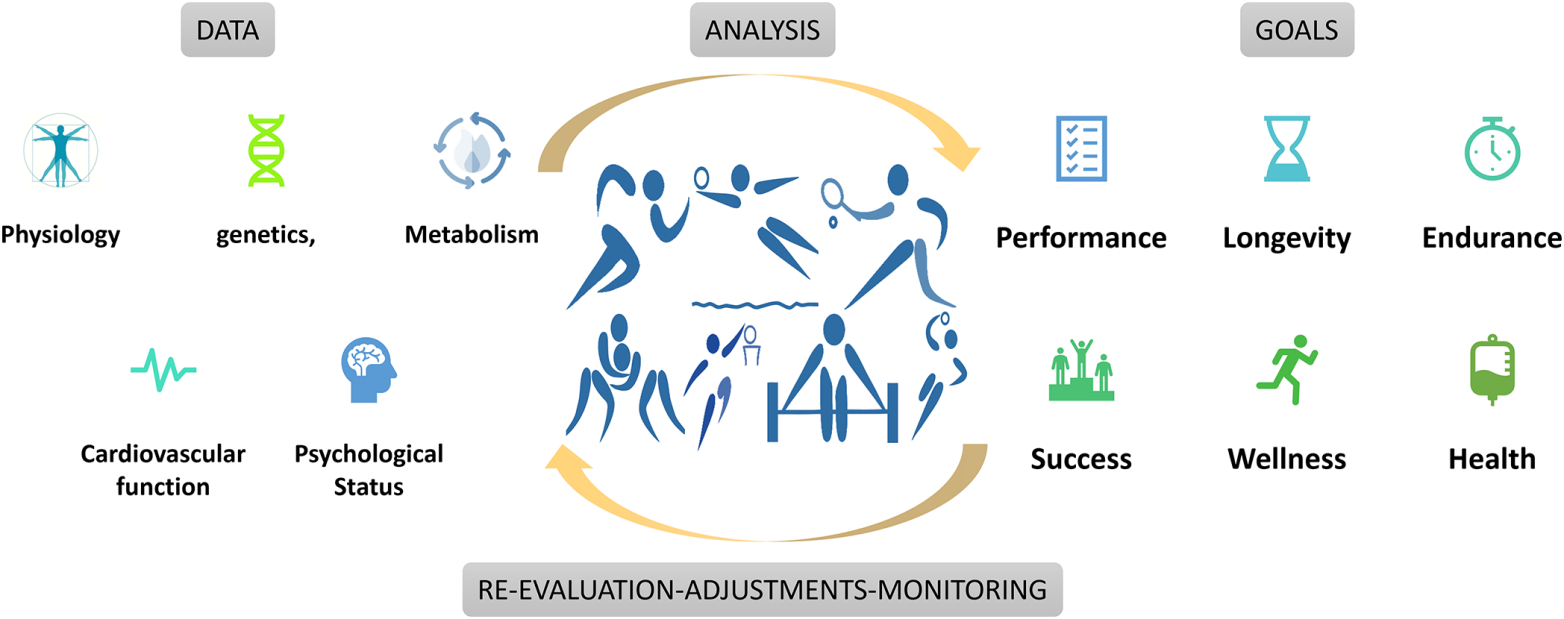
Data analysis and program adaptation based on athlete goals.

Precision or Personalized medicine aims to provide prevention and treatment strategies for defined groups of individuals, as accepted by the EU Health Ministers in their Council conclusions in December 2015 (9). Expanding this approach to athletes, a comprehensive view of biological, nutritional, and pathophysiological status could be described, attenuated, and enhanced (10). Leveraging advanced technologies and follow-ups enables tailored approaches to promote sustained health, endurance, and athletic longevity (Figure 1). The current work introduces a prospective methodological framework encompassing targeted analysis of telomere dynamics, standardized genotyping/phenotypic profiling, biochemical tests, metabolomics, essential elements assessment, echocardiography, and burnout evaluation. These domains hold immense promise in leading in a new era in sports medicine, offering an evidence-based, holistic, and personalized approach for enhancing athletic performance.

### 1.1 Telomere analysis

Telomeres are repetitive (TTAGGG)n sequences of DNA without translational value, allied with specific proteins (shelterin complex) to form single-stranded structures called telomere loops at chromosomes' ends of eukaryotic cells (11). Their role is to preserve genetic information, maintain chromosome integrity and protect against degradation, recombination, or fusion. With each cell division chromosomes naturally lose a small section of their telomeres. This reduction continues until it reaches a certain point (Hayflick limit) after which DNA mechanisms are activated leading to cellular senescence or apoptosis (12). The abundance and shortening rate of telomeres are strong determinants of cell homeostasis and altered by oxidative stress and lifestyle factors such as diet, exercise, stress, smoking etc. (13). The length and rate of shortening of telomeres are characteristic of a person's biological age which does not always coincide with their chronological age (14). Telomere shortening is linked to chronic disorders (e.g., cardiovascular, neurodegenerative, autoimmune etc.) and is also observed in athletes who abuse performance-enhancing agents (15–17). Telomere analysis provides insights into cellular aging, both systemic and at a tissue/organ level, by measuring telomere length and telomerase activity (18). Telomerase is an enzyme that maintains telomere length and thus preserves cellular integrity. Under normal conditions, enhanced telomerase activity has been linked to better telomere maintenance and potentially slower cellular aging (19, 20). Research data have shown that high levels of physical activity and regular exercise may enhance telomerase activity (21, 22). Understanding athletes' telomere dynamics can uncover associations of their biological age as to their cellular health, resilience to training-induced stress and predicting their longevity (21, 23). Athletes seem to have longer telomeres than their age-matched non-athletes. This is associated with lower oxidative stress biomarkers and chronic inflammation as well as up-regulated expression for shelterin and telomerase activity (24–26). Moreover, a protective impact of exercise has been shown in older endurance athletes who display adequate telomere length and up-regulated telomere-related genes (27). Additionally, younger athletes engaged in higher-intensity sports seem to exhibit elevated levels of pro- and anti-inflammatory cytokines, which contribute to telomere maintenance (28). Although further research is needed to understand these complex relationships, incorporating telomere analysis can provide personalized strategies to optimize training programs towards athletic longevity.

### 1.2 Genotype/phenotype analysis

Systems biology provides a new perspective in sports medicine for studying athletes' genotypes, epigenetics, and phenotypic profiles (29–32). Polymorphisms can be associated with athletic features, such as endurance, strength or ability to generate force, power, aerobic capacity (VO_2_ max), flexibility, neuromuscular coordination, psychological responses, injury predisposition, post-workout stress recovery needs, and other related factors (10). Analyzing an athlete's genetics and physical traits may reveal insights into genetic factors influencing athletic abilities, injury susceptibility, and potential underlying conditions. This information can highlight inherent strengths while identifying areas needing greater attention for improvement. To date, over 250 genetic markers have been identified with potential links to sport-related traits. Among these, 128 markers have been examined by multiple studies, but data are still limited, and further research is required to fully understand the mechanistic details (33). Nevertheless, exploitation of these data assists as a personalized guide, indicating the effort required for an athlete to achieve peak performance, without imposing limitations (34). Phenotypic analysis complements genotyping by assessing apparent traits and characteristics resulted from genetic variations related to physical attributes and functional capacities (35). For example, the alpha-actinin-3 protein, encoded by the ACTN3 gene located on chromosome 11q13.1, is predominantly expressed in fast, type II muscle fibers, contributing to the generation of fast and powerful muscle contractions. A specific gene variation (rs1815739) involves a C-to-T transition in exon 16. This results in the replacement of an arginine with a stop codon at amino acid 577 (R577X), leading to the absence of functional α-actinin-3 protein. An over-representation of the ACTN3 RR genotype and an under-representation of the ACTN3 XX genotype in strength/sprint athletes is often observed (36).

Genetic testing can also contribute in early diagnosis of potential pathophysiological factors and genetic predispositions for specific injuries and conditions (37). For instance, the growth differentiation factor 5 (GDF5) protein, which plays a role in bone, muscle, tendon growth and maintenance, has three gene variants (CC, CT, TT). T alleles are associated with lower GDF5 protein levels and a higher frequency of osteoarthritis (38). Moreover, genetic variations may reveal risk for cardiovascular (i.e., arrhythmias and sudden heart death) or other disorders (39). For example, myosin-binding protein C3 (MYBPC3) gene appear to influence endurance and performance among elite athletes, although certain variants have been associated with hypertrophic cardiomyopathy (40). Although further research is needed (i.e., environmental impact), these technologies have the potential for personalized interventions, including tailored training, recovery, injury prevention or medical monitoring (41).

### 1.3 Biochemical tests

Biochemical tests are the most employed method for monitoring athletes' health and performance (1). Blood biomarkers offer a wealth of information about an athlete's metabolic processes, immune system response, and nutritional status (42). For example, red blood cell or hematocrit values are essential indicators of oxygen-carrying capacity and overall blood volume (43). White blood cell count, C-reactive protein, and erythrocyte sedimentation rate offer insights into an athlete's immune system and stress response. This enables early detection of excessive inflammation, helping prevent overtraining syndrome and optimize recovery for reduced injury risks (44, 45). Electrolyte levels, liver enzymes (alanine transaminase, aspartate transaminase), and kidney function markers (creatinine, blood urea nitrogen), offer information about an athlete's metabolic health and organ function (46). Blood lipid profiles, and cholesterol levels (low- or high-density lipoproteins, triglycerides), provide insights as to an athlete's cardiovascular health and lipid metabolism (47). Creatine kinase, myoglobin, and markers of oxidative stress (e.g., malondialdehyde) help assess the extent of muscle tissue breakdown and the effectiveness of recovery interventions (48). In addition, quantification of vitamins, minerals, and hormone levels provide information about an athlete's nutritional needs and potential deficiencies (49).

### 1.4 Metabolomics

Metabolomics refers to analysis of low-molecular weight metabolic products (<10–15 kDa), known as the metabolome and their intricate network of reactions and pathways. Metabolomics enhance our understanding of cellular metabolism, disease mechanisms, and the impact of interventions or external factors such as exercise programs (50). Parameters such as lactate, glucose, and free fatty acids offer useful information about an athlete's energy production, utilization, and substrate preferences during exercise (51). Elevated lactate levels may indicate the onset of anaerobic metabolism and help adjust training intensity accordingly (52). Changes in glucose levels can indicate the utilization of carbohydrates while variations in free fatty acids and ketone bodies, can indicate enhanced fat utilization due to endurance training (53). Alterations in amino acid profiles can reveal the impact of training on protein turnover and the utilization of amino acids for energy or muscle repair processes (54). Metabolomics can identify specific metabolic signatures linked to different training approaches. For instance, it can unveil unique metabolite patterns for high-intensity interval training compared to steady-state endurance training (55). These signatures inform tailored training and nutrition plans, aligning with each athlete's unique metabolic needs. Integration with other “omics” disciplines can locate metabolic pathways influenced by specific genes or proteins, offering further insights for personalized approaches to training and nutrition (29).

### 1.5 Essential elements

Essential elements (i.e., minerals and trace elements) are critical components in evaluating the micronutrient status of athletes. They contribute to physiological processes in locomotor (musculoskeletal), nervous, and immune system factions as well as for metabolic processes (56). Quantifying essential element levels enables the evaluation of nutritional status and the application of interventions to optimize performance (57). For example, iron deficiency can lead to reduced oxygen-carrying capacity and impaired athletic performance (58). Measuring serum ferritin levels helps assess iron status, guiding targeted iron supplementation or dietary adjustments as needed (59). Calcium levels in blood or bone density measurements can ensure adequate calcium intake and implement strategies to support bone health and muscle function (60). Sufficient Magnesium levels will reduce risk for muscle cramps, impaired muscle function, and reduced exercise performance especially during demanding practice sessions or in game (61). Zinc's low levels can compromise immune function, leading to increased susceptibility to infections and impaired recovery thus incorporation of zinc-rich foods or supplements in athletes' diet is necessary (62). Selenium deficiencies can disrupt immune and thyroid hormone balance impacting energy metabolism and overall athletic performance (63).

### 1.6 Echocardiography

Echocardiography, a non-invasive ultrasound technique, offers detailed heart structure and function images, evaluating crucial cardiac parameters for athletic performance. Athletes often exhibit adaptations in cardiac size and shape due to the demands of their training (64). There is strong evidence that anabolic agents' administration, which is often encountered in athletes, could induce a detrimental effect to heart diastolic function, which should be closely monitored (65). Echocardiography assesses whether these adaptations are within normal physiological ranges or indicate potential underlying cardiac issues warranting further investigation. For young athletes, the European Society of Cardiology recommends a three-step process: evaluating family and personal history, conducting a physical examination, and performing a 12-lead electrocardiogram (66). Echocardiography also evaluates pumping efficiency and the delivery of oxygenated blood to body tissues, examining parameters like ejection fraction, cardiac output, and myocardial contractility (67). Valvular abnormalities, such as regurgitation or stenosis, can have a significant impact on cardiac performance and their detection is feasible through echocardiography (68). Also, the presence of fibrosis or scarring can be identified and evaluated (69). Overall, echocardiography identifies potential cardiac issues or prior injuries that may pose health risk whereas for healthy athletes, regular echocardiography maximizes cardiovascular function and supports long-term health (70).

### 1.7 Burnout tests

Assessing an athlete's well-being and performance is crucial due to the potential impact of emotional and physical exhaustion on their health (71). Tests such as the Maslach Burnout Inventory (MBI) gauge burnout symptoms, allowing early intervention to prevent worsening. The MBI evaluates emotional exhaustion, depersonalization, and reduced personal accomplishment offering valuable insights into an athlete's well-being (72). Physical burnout tests, examining signs like chronic fatigue and reduced performance, complement these assessments (73). Physiological markers like heart rate variability and cortisol levels alongside these tests offer insights of the athlete's physical stress levels and potential burnout risk. Questionnaires or interviews can further explore contributing factors like training load, recovery, support systems, and stressors (74). Integrating burnout assessments into athlete care plans is vital to prevent exhaustion's effects and ensure long-term success, involving adjustments in training plans, enhanced recovery strategies, psychological support, and fostering a supportive team environment.

## 2 An integrated perspective for athletes

**Figure 2:**
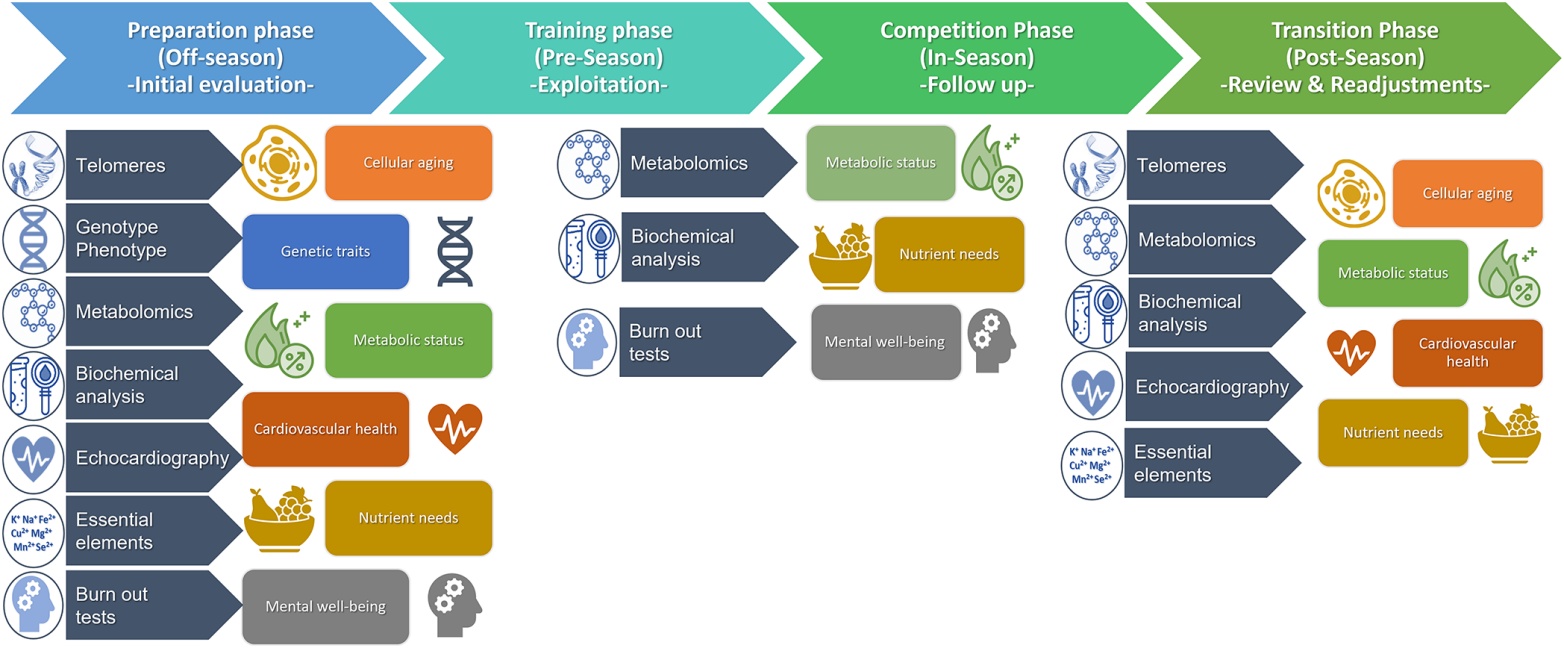
An integrated methodological approach for assessment of athletes’ health status, well-being, and longevity throughout a typical 1-season timeline.

The integration of -omic and telomere technologies, coupled with conventional approaches for biochemical analysis, medical imaging, and mental assessments, could potentially constitute a novel iterative process in enhancing athletic performance within an athletic season. Each component offers crucial insights, contributing uniquely to a comprehensive understanding of athletes' health and wellness (Figure 2). Initially, during the preparation phase (off-season), an initial evaluation of medical history, physical examination, and lifestyle establishes an athlete's health baseline and goals followed by analysis of biological matrices. Blood and saliva samples are used for telomere analysis to assess cellular aging. Blood and urine samples are processed for biochemical tests and quantification of metabolomics biomarkers. Genotype/Phenotype performed in saliva samples to identify specific genes and genetic variations related to athletic traits, injury susceptibility, or underlying health conditions. Micronutrient levels and essential elements are quantified in hair samples to assess nutritional status. Echocardiography examines cardiac structure, function, and vascular health, crucial for young athletes to detect early heart abnormalities. Mental well-being assesse emotional status, performance, and motivation. Integration of these data leads to personalized insights of athlete's cellular aging, genetic traits, metabolic status, cardiovascular health, nutrient needs, and mental resilience. Afterwards, tailored interventions including training, nutrition, recovery, and psychological support, are developed based on individual profiles. Regular follow-up assessments during pre-season and in-season timeframes monitor progress, track changes, and make necessary adjustments to interventions that ensures ongoing optimization of the athlete's personalized goals and promotes optimal performance, injury prevention, and long-term athletic success while prioritizing the athlete's overall health and well-being. Finally, post-season re-evaluation of wellness, longevity, and overall health establishes new baselines before the rest period ahead of the new season.

## 3 Discussion

The combination of the presented methodologies for assessment of athletes' performance and longevity is based on their interrelation and impact on an athlete's health, wellness, and longevity. Each assessment offers distinct insights, informing the interpretation and management of other parameters. This creates a comprehensive and integrated approach to athlete evaluation. For example, telomere analysis emphasizes well-being and longevity, while biochemical tests center on athletic performance.

Telomere dynamics are influenced by genetic and environmental factors including nutritional intake and dietary habits. Genotype/phenotype analysis reveals genetic variations associated not only with metabolism but also expands to telomere length and integrity (75). Thus, an athlete's genetic makeup offers insights into their cellular aging pattern and potential effects on performance and longevity. Meanwhile, telomere dynamics can unveil molecular mechanisms related to specific athletic traits or susceptibility to age-related health conditions (21, 24, 25). Furthermore, the phenotypic expression of genetic traits, as well as telomere length and integrity, are influenced by epigenetic regulation affected by lifestyle, nutrition, and environmental factors (31). This interplay between genetic and epigenetic factors contributes to the complex phenotypic expression observed in athletes and underscores the importance of considering both genetic and epigenetic influences when analyzing athletic performance (25). It should always be considered that genetic predisposition is not the sole determinant for athletes' success, but it comes along with the impact of environmental and epigenetic variables that outline the final phenotype (32, 34). In this regard, while genetic analysis is a one-time necessity, the ongoing assessment of phenotypic expression is feasible for specific traits (i.e., metabolites) throughout an athlete's career. Furthermore, socio-economic factors, personal determination, and well-structured athletic programs with expert coaching and scientific support play crucial roles in shaping an athlete's development and enabling them to achieve their goals (76). Biochemical tests assist in analysis of athletes' overall health. Biochemical traits as well as metabolomics are also influenced from genotype/phenotype characteristics, environmental factors, lifestyle, and training (77). Metabolomics enriches biomarker data from biochemical tests, providing a comprehensive understanding of metabolic adaptations, energy production, and potential indicators of fatigue or overtraining (78). Essential elements can unveil nutrient deficiencies or imbalances affecting an athlete's metabolic state and organ function. This, in turn, can influence biochemical tests, metabolomics data, and the cellular aging process indicated by telomeres (79). Echocardiography assesses cardiac features, offering insights into cardiovascular performance. It also contextualizes metabolic adaptations observed in metabolomics and biochemical tests, aiding in the diagnosis of potential pathophysiological factors linked to genetic predispositions for specific conditions (70). Mental and physical endurance impact an athlete's ability to sustain long-term training and performance excellence (73). Burnout test results reveal psychological stressors’ impact on metabolic, cardiovascular, and cellular processes. While data is inconclusive, biochemical, metabolomics, or telomere analysis might be linked to potential burnout symptoms or mental fatigue in athletes (80).

In the context of the discussed components, it should be added that nutritional programs play a pivotal role in athletes health (79). Exercise is linked to localized and systemic oxidative stress and inflammation, both of which correlate with muscle fatigue and reduced performance. Ergogenic and antioxidant effects of dietary interventions could alleviate detrimental side-effects of training, as identified by a multi-targeted testing methodology hereby discussed (81). By synthesizing insights from aforementioned components, tailored nutritional interventions can be developed to address individual needs and goals. These interventions offer strategies for fueling, timing, and supplementation to support energy, recovery, and immune function. They also address essential element requirements, ensuring athletes receive vital micronutrients (82). Moreover, they consider individual variations in genetic profiles, metabolomics, nutrient utilization, and dietary preferences or restrictions (83). For instance, nutrients, like folate, vitamins B12, and B6 impact epigenetic processes and gene expression patterns (84). Customized nutritional strategies, informed by an athlete's genotype/phenotype and understanding of nutrition's influence on epigenetics, can optimize gene expression profiles and enhance specific athletic traits. Well-designed nutritional programs, integrated into the assessment framework, contribute to performance enhancement, overall health, and athlete longevity in their respective sports. It's important to note that genetic modification (gene doping) and illegal supplement use are beyond the scope of this approach, as they are prohibited and unethical practices in sports (85). Lastly, acknowledging potential challenges in interpreting personalized interventions using clinical, physiological, and biomedical information, refining methodologies on data analysis will be key to overcoming these obstacles (86–89).

## Data Availability

The original contributions presented in the study are included in the article/Supplementary Material, further inquiries can be directed to the corresponding author.
